# The Influencing Effect of Tourism Economy on Green Development Efficiency in the Yangtze River Delta

**DOI:** 10.3390/ijerph20021072

**Published:** 2023-01-07

**Authors:** Meijuan Hu, Zaijun Li, Bing Hou

**Affiliations:** 1 School of Tourism and Cuisine, Yangzhou University, Yangzhou 225127, China; 2 Institute of Tourism Culture, Yangzhou University, Yangzhou 225127, China; 3 Research Institute of Central Jiangsu Development, Yangzhou University, Yangzhou 225127, China; 4 Institute of the Grand Canal Research, Yangzhou University, Yangzhou 225009, China

**Keywords:** green development efficiency, tourism development, panel vector autoregressive model (PVAR), Super-EBM model, Yangtze River Delta region

## Abstract

In the context of ecological priority and green development strategy, accelerating the upgrading of tourism structure and promoting the development of ecotourism is an important guarantee to achieve green and low-carbon economic growth and high-quality development. On the basis of constructing comprehensive evaluation indicators of tourism development (TD) and green development efficiency (GDE), this study analyzed the impulse response relationship between TD and GDE and the impact effect of TD on GDE in the Yangtze River Delta region from 2000–2018. Findings showed that: (1) During the study period, TD generally exhibited a W-shaped fluctuating upward trend and GDE showed a staggered evolution of upward and downward fluctuations, while both regional gaps of TD and GDE continued to decrease. (2) Most cities had made a leap from low to medium, high, and higher levels of tourism development, with tourism development levels decreasing along the Yangtze River basin to the north and south of the delta. The overall green development efficiency was relatively low, showing a spatial pattern of high value in the southern delta and low value in the northwest delta. (3) There was a one-way Granger causality of TD on GDE, and the impact of TD on GDE showed a significant positive cumulative effect. (4) TD exhibited a significant inverted U-shaped impact on GDE. The economic development level and government intervention had a significant positive impact on GDE. The proportion of secondary industry, energy consumption intensity, and foreign direct investment had a significant negative driving effect on GDE. While the impact of environmental regulation on GDE was insignificant positive. This study has great practical significance to alleviate the problems of urban resources and environment, and to realize a green economy and high-quality life.

## 1. Introduction

As China’s economy shifts from the stage of rapid growth to the stage of high-quality development, it is required to shift from the extensive stage of high-speed growth relying on increasing material resource consumption to the stage of high-quality development relying on technological progress and improving the quality of workers [1,2,3]. Under the appeal of ecological priority and green development, green is considered the bright color of the current economic and social construction and development, green development has been promoted as an important strategy under the new normal of the economy, and the concept of green development has become the main theme of national economic and social development in the new period, and improving the efficiency of green development has become the “golden key” to promote high-quality economic development, and an inevitable choice to break through resource and environmental constraints, upgrade industrial structures, and achieve sustainable development [4,5,6]. Therefore, it is necessary to promote the transformation of the urban green economy and accelerate the pace of urban green development.

In recent years, the scale of the tourism economy has been expanding and the status of tourism has been gradually strengthened, with a comprehensive contribution rate of more than 10% to the social economy and an important role in the effective improvement of the urban ecological environment [7]. Thus, tourism is often referred to as a smokeless industry and green industry with resource-saving and environment-friendly properties [8]. However, it is worth noting that tourism is also a major contributor to carbon consumption and emissions, and its development consumes the natural resources of destinations and generates pollutants such as waste gas, wastewater, and waste solids [9,10]. Therefore, the tourism industry has an important mission to reduce pollution and carbon in the trend of green development and ecological civilization construction. As one of the regions with the fastest urbanization and the most developed heavy industries in China, the Yangtze River Delta region is confronting the problems of heavy industrial structures, coal-fired energy structures, and un-decoupled economic growth and environmental pollution emissions, which makes the construction of ecological civilization encounter the severe situation of overlapping many contradictions such as serious ecological degradation, deterioration of residents’ quality of life, and massive space encroachment. The tourism industry in the Yangtze River Delta region ranks among the highest levels of development in China and has become an important sector for creating material wealth and optimizing industrial restructuring. Under the background of ecological priority and green development strategy, the arguments about the green industry attributes of the tourism economy mostly focus on whether the tourism economy itself is green or not, lacking a scientific understanding of the green externalities of the tourism economy, namely whether tourism can contribute to the overall green development of the region from a macro perspective. Therefore, re-examining the impact of the urban tourism economy on green development efficiency, exploring the spatio-temporal dynamic relationship between the tourism economy on green development efficiency, and constructing a measurement model of the impact of the tourism economy on green development efficiency are of great theoretical and practical significance for improving the theory of green growth and regulating the development path of Yangtze River Delta cities.

## 2. Literature Review

Green development efficiency is a measure of the development capacity of the economic-social-environmental system under the constraints of ecological environmental capacity and resource carrying capacity, and an important manifestation of the coordinated and sustainable development of the human–land relationship under the guidance of the green development concept [11,12]. In the context of high-quality development, green development efficiency has received wide attention as an important tool to measure the effectiveness of high-quality development and ecological civilization construction. The concept of green development was first introduced by David Pierce in 1989 as a form of affordable economic growth [13]. With the gradual deepening of research, the concepts of green economy, circular economy, green transformation, and low-carbon development have emerged one after another [14,15]. Loiseau et al. (2016) analyzed the connotation of the concept of green development from economic, environmental, and social dimensions and developed a more systematic and hierarchical relation among circular economy, green economy, and bioeconomy [16]. In terms of empirical research, relevant studies have investigated green development efficiency from multi-scale and multi-mechanism perspectives, mainly focusing on the spatial and temporal patterns and influencing factors of regional green development efficiency, the relationship between environmental regulation and green development efficiency, the promotion effect of technological innovation and industrial agglomeration on green development efficiency [17,18,19,20]. However, the role played by green industries such as tourism in the green development process was less analyzed.

International organizations generally regard tourism as an important industry to promote the development of the global economy towards green transformation, which has prompted academics to re-examine the green attributes of tourism, especially theoretical and empirical studies on the green development effects of the tourism economy. Some researchers began to analyze the impact of tourism development on green growth in terms of quantitative model results or qualitative explanations. For example, Marsiglio (2015) used a dynamic economic model to deductively analyze the impact of economic activities in island-based tourist destinations on economic growth and the environment, arguing that tourism could contribute to regional energy conservation and economic growth, and was an enabler of regional green economic growth [21]. Gupta and Dutta (2017) found that tourism could increase national wealth in underdeveloped areas, but it could also destroy the tourism ecosystem under a new steady-state equilibrium [22]. Pan et al. (2018) argued that there was an interaction between tourism and economic, social, ecological, and cultural sustainability, and that tourism could promote the transformation of the green economic system [23]. Feng focused on the material balance model, made a theoretical discussion on the mechanism of how global tourism promotes green development, and found that the tourism economy affects regional economic growth and environmental quality levels through direct or indirect effects [24].

Based on the empirical framework of green impact, scholars follow the research paradigm of economics to examine the economic and environmental externalities of tourism. However, relevant studies are mostly on large and medium scales, and it is difficult to form a consensus due to the different samples and time periods of the studies. Some researchers found that tourism contributed to the country’s economic growth and played an important functional role in carbon emission reduction, and concluded that tourism had significant green attributes [25,26,27]. While others conducted empirical analyses of the BRICS countries and found that tourism not only promoted regional economic development, but also increased total regional carbon emissions, which in turn caused a decline in the environmental quality of the destination [28,29,30,31]. Besides, Liu et al. (2022) found that there was an inverted U-shaped relationship between tourism and environmental pollution, and carbon emissions increased with the rapid development of tourism and then tended to decline after a limit [32]. Research on the tourism economy’s impact on China’s green development is relatively scarce and focused on a large scale. For example, Tong et al. (2021) analyzed the spatial spillover effect of the tourism economy on the green development efficiency of 284 Chinese cities from 2005 to 2016 and found that the tourism economy exhibited a significant impact on green efficiency change and green technology change, but had an insignificant positive spatial spillover effect on green total factor productivity [33]. By constructing a PSM-DID model, Li et al. (2020) found that the creation of national ecotourism demonstration zones in cities contributed to increasing local per capita income, strengthening the effectiveness of environmental regulation, and promoting regional green development [34].

Although a large number of studies have been conducted around TD and GDE, there are still some gaps to extend. First, related studies generally focused on the separate impact of tourism development on the regional economy or regional environment, resulting in inconsistent findings. While green development efficiency emphasizes the harmonious growth of the economy and the environment, thus, it is worth deeply revealing the spatio-temporal interaction mechanism between TD and GDE and analyzing the impact of TD on GDE, which can enrich the evaluation of the environmental impact effect of tourism development. Second, current studies generally used a single or a few aggregated indices to quantify TD and GDE, ignoring the comprehensive features of TD and GDE. Third, relevant studies on the effect of TD on GDE focused on provincial units, and there were few studies on the impulse response and driving effect between TD and GDE at the city level. Therefore, on the basis of constructing the evaluation index of green development efficiency and tourism development, this study analyzed the spatio-temporal interaction between tourism development and green development efficiency and the impact of tourism development on green development efficiency of 41 cities in the Yangtze River Delta region from 2000–2018.

## 3. Study Area, Methods and Data

### Study Area

**Figure 1:**
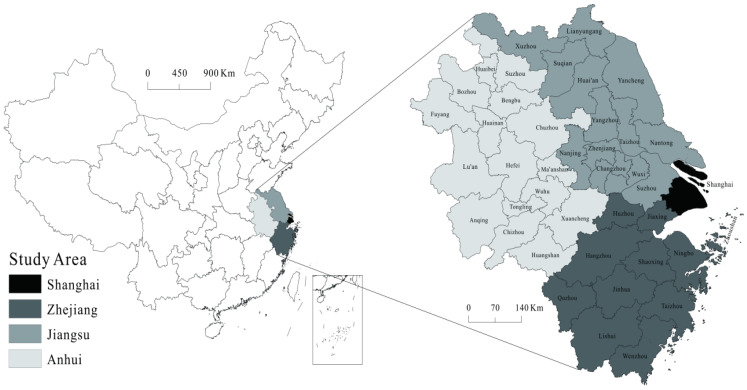
The study area.

This study takes the Yangtze River Delta region as the study unit, including Jiangsu Province, Zhejiang Province, Anhui Province, and Shanghai City (Figure 1). The Yangtze River Delta region is an important intersection of the “Belt and Road” construction and the Yangtze River Economic Belt and is also the economic section with the largest economic volume, the greatest development potential, the highest degree of openness, and the strongest innovation capacity in China. With the regional integration of the Yangtze River Delta rising to the level of national strategy in 2008, the degree of regional integration development is also gradually deepening. However, the Yangtze River Delta region has long relied on high input, high resource consumption, and high environmental pollution in exchange for a high-speed economic growth mode, and is facing severe ecological and environmental pressures such as severe resource shortage and environmental pollution. Therefore, it is important to promote green industry transformation to achieve high-quality development.

## 4. Methods

### 4.1. PVAR Model

The panel vector auto regression (PVAR) model was proposed by Sims, which uses the lagged values of variable systems as endogenous variables to analyze and predict the dynamic interrelationship, impact intensity, positive and negative shocks, and duration between the time series variable systems [35,36]. It can be expressed as follows:

(1) $$Y_{it}=a_{{}_{0t}}+\sum_{i=1}^{m}a_{{}_{li}}Y_{it-1}+f_{i}+d_{c,t}+u_{it}$$

where *i* and *t* denote the sample unit and year, respectively; $Y_{it}$ are the vectors of tourism development and green growth efficiency; *m* is the lag order of the model; *f_i_* represents the individual fixed effect; $a_{0t}$ is the intercept term; $a_{1t}$ is the influencing coefficient of the interaction effects; $d_{c,t}$ is the time-fixed effect; $u_{it}$ is the disturbance term. Before estimating this model, the following three steps need to be performed. Firstly, the unit root test is used to test the time stationarity of tourism development and green growth efficiency. Secondly, the Johansen co-integration test is applied to verify the long-run equilibrium relationship between the two systems. Thirdly, the PVAR model is estimated to analyze the mutual impact intensity between the two systems.

### 4.2. Super Epsilon-Based Measure (EBM) Model

The green growth efficiency measured by the traditional DEA model from the radial perspective is prone to bias due to the neglect of the slackness between the desired and undesired outputs. Hence, Tone and Tsutsui (2010) further proposed a non-radial hyper EBM-DEA model, which comprehensively considers expected and unexpected outputs, and can effectively solve the problem that efficiency values cannot be sorted [37]. This study uses the Super-EBM model to measure the efficiency of green development, and measures the efficiency by the ratio of input, the minimization of undesired output, and the maximization of expected output, which fully reflects the multiple green goals such as economic growth, environmental friendliness, energy conservation and carbon reduction [38,39]. The formula is as follows:

(2) $$\min_{\rho *}=\frac{\frac{1}{M+N}(\sum_{m=1}^{M}\frac{x_{}^{-t^{0}}}{x_{mj^{0}}^{t^{0}}}+\sum_{n=1}^{N}\frac{e_{}^{-t^{0}}}{e_{nj^{0}}^{t^{0}}})}{1+\frac{1}{Q+H}(\sum_{q=1}^{Q}\frac{y_{}^{-t^{0}}}{y_{qj^{0}}^{t^{0}}}+\sum_{n=1}^{N}\frac{b_{}^{-t^{0}}}{b_{hj^{0}}^{t^{0}}})}$$

(3) $$x^{-t^{0}}\geq \sum_{t=1}^{20}\sum_{j=1,\neq k}^{41}x_{mj}^{t}\lambda_{j}^{t},m=1,\ldots ,M;\;e^{-t^{0}}\geq \sum_{t=1}^{20}\sum_{j=1,\neq k}^{41}e_{nj}^{t}\lambda_{j}^{t},n=1,\ldots ,N$$

(4) $$y^{-t^{0}}\leq \sum_{t=1}^{20}\sum_{j=1,\neq k}^{41}y_{qj}^{t}\lambda_{j}^{t},q=1,\ldots ,Q;\;b^{-t^{0}}\geq \sum_{t=1}^{20}\sum_{j=1,\neq k}^{41}b_{nj}^{t}\lambda_{j}^{t},h=1,\ldots ,H$$

(5) $$\sum_{t=1}^{T}{\sum_{j=1}^{J}\lambda }_{j}^{t}=1;\;\lambda_{j}^{t}>0;\;x^{-t^{0}}\geq x_{k}^{-t^{0}};e^{-t^{0}}\geq e_{k}^{-t^{0}};y^{-t^{0}}\geq y_{k}^{-t^{0}};b^{-t^{0}}\geq b_{k}^{-t^{0}}$$

where: $s_{m}^{-x},s_{n}^{-e},s_{q}^{+y},s_{h}^{-b}$$m_{k}^{}$ are the slack variables for non-energy input, energy input, expected output, and undesired output, respectively.

### 4.3. System Dynamic Panel Regression Model (SGMM)

The ordinary least regression (OLS) model can inevitably lead to some bias estimation due to the endogeneity problem of the model. To avoid this issue, a two-step system generalized method of moments (SGMM) model based on Arellano and Bover (1995) and Blundell and Bond (1998) is chosen to explore the dynamic impact effect of TD on GDE. Due to the inertia of the green economy, the first-order lag term of green development efficiency is introduced. Moreover, to test the nonlinear relationship between TD and GDE, the quadratic term of tourism development is introduced. The dynamic panel model is constructed as follows [40,41]:

(6) $$GDE=\mu_{i}+\beta_{1}GDE_{it-1}+\beta_{2}TD_{it}+\beta_{3}TD_{it}^{2}+\theta_{i}x_{it}+\varepsilon_{it}$$

where i represents the city unit, t represents year, $x_{it}$ is a series of control variables, $\theta_{it}$ is the corresponding coefficient of the control variables, $GDE_{it-1}$ is lagged term of green development efficiency, $\mu_{i}$ is the individual fixed effect of city, and $\varepsilon_{it}$ is the random disturbance term. It should be noted that in order to further verify the validity of SGMM estimation results, the mixed OLS estimation and fixed effect model are also estimated.

### 4.4. The Construction of Evaluation Index System

The green development efficiency was mainly measured from the perspective of input-output based on green total factor productivity, which effectively integrated the benefits of economic growth and environmental protection [42,43]. Among them, capital, labor, and technology were selected as non-resource inputs, and energy and land resource consumption was selected as resource input. In terms of outputs, economic benefit, social benefit, and environmental benefit indexes were selected as desirable outputs, and environmental pollution indexes were selected as undesirable output (Table 1). Tourism development was mainly evaluated from three aspects in Table 2, including scale, quality, and structure of the tourism industry [44,45,46].

### 4.5. Data Sources

A total of 41 cities in the Yangtze River Delta region were selected as research units, and the research period was defined from 2000 to 2018 due to data availability. Related data indicators were sorted and calculated: Shanghai Statistical Yearbook (2001–2019), Zhejiang Statistical Yearbook (2001–2019), Jiangsu Statistical Yearbook (2001–2019), Anhui Statistical Yearbook (2001–2019), China City Statistical Yearbook (2001–2019), China Environmental Statistical Yearbook (2001–2019), Statistical Bulletin on National Economic and Social Development of Cities and Statistical Bulletin on Urban Health from 2000–2018. In order to avoid the influence of extreme differences in indicators on the results, all indicators were processed by per capita, ground average, or percentage. Some economic data such as GDP, consumption, and income were converted based on the year 2000.

## 5. Results

### 5.1. The Spatial and Temporal Evolution Characteristics of Urban Tourism Development and Green Development Efficiency

**Figure 2:**
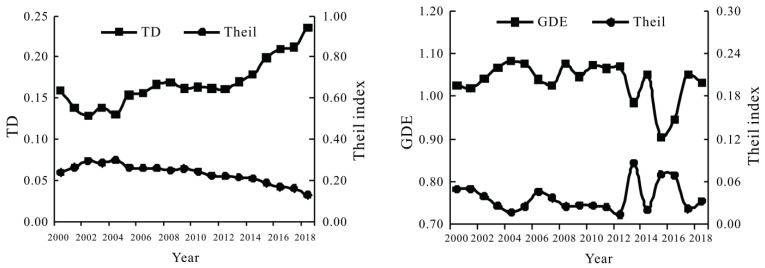
The evolution trend of TD and GDE in the Yangtze River Delta region from 2000 to 2018.

The Super-EBM model and entropy-weighted method were respectively used to calculate the GDE and TD of 41 cities in the Yangtze River Delta region from 2000 to 2018, and then the Theil index was used to calculate the regional differences (Figure 2). As shown in Figure 2, from 2000 to 2018, the tourism development level in the Yangtze River Delta region generally showed fluctuating upward trend. The difference in regional tourism development showed an inverted U-shaped evolution trend. The green development efficiency and its regional difference exhibited a staggered evolution trend of high efficiency and low difference or low efficiency and high difference. Specifically, the evolution process could be divided into the following three stages. The first stage (2000–2004) was the initial rise of green growth efficiency. The green development efficiency increased from 1.025 in 2000 to 1.082 in 2004, with an average annual growth rate of 5.56%. The second stage (2004–2015) was the ebb and flow of green development efficiency. There presented a downward-upward W-shaped fluctuation trend from 2004 to 2010 and a downward-upward-downward inverted N-shaped fluctuation trend from 2010 to 2015. While the regional differences showed an inverted V-shape decrease pattern from 2004 to 2012 and an N-shaped increase pattern from 2010 to 2015. In the third stage (2015–2018), the green development efficiency presented an inverted V-shaped upward trend, and the Theil index of green development efficiency showed a downward trend.

**Figure 3:**
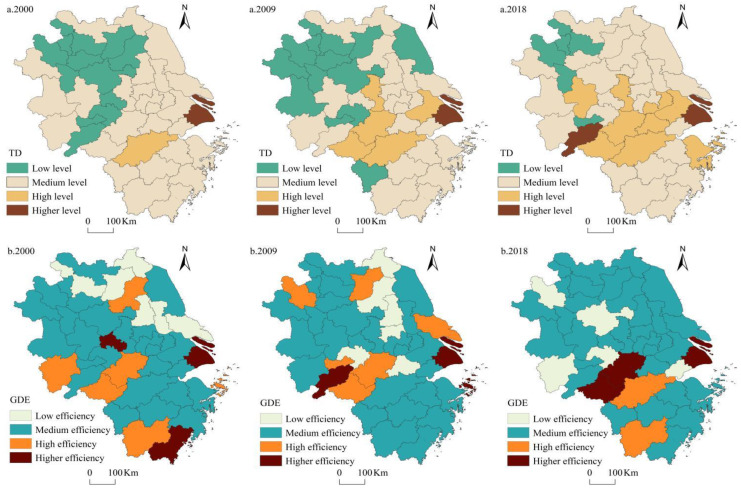
Spatial pattern of TD and GDE in the Yangtze River Delta region. Note: (**a**) represents the development level of TD in 2000, 2009 and 2018; (**b**) represents the development level of GDE in 2000, 2009 and 2018.

In order to depict the spatial differentiation characteristics of urban tourism development, the interquartile spatial visualization was carried out using 0.1, 0.3, and 0.5 as critical values to classify tourism development into four types, namely, low, medium, high, and higher levels (Figure 3a). As shown in Figure 3a, most cities had achieved a significant increase in the level of tourism development since 2000, with an increase in the number of cities with high and higher levels of tourism development increasing and a decrease in the number of cities with low levels of tourism development. There were large spatial differences in the development of urban tourism economy, showing a pattern of “high in the southeast and low in the northwest”. Shanghai had always kept a higher level of tourism economy, far ahead of other cities. Hangzhou had always focused on the construction of a livable and touristy city, and the level of tourism economy had stabilized at a high-level echelon. The tourism development level of Suzhou, Huaibei, Bozhou, Huainan, Fuyang, and Tongling in Anhui Province had been low for a long time, and it was difficult to jump because of the relative lack of tourism resources and the lack of momentum of capital introduction. Hefei, Nanjing, Suzhou, Huangshan, Wuxi, and other cities had risen to a high level of tourism development echelon due to their developed economy levels and rich tourism resources. In terms of provincial scale, Shanghai, Zhejiang, and Jiangsu all achieved medium and above levels of tourism development in 2018, with only some cities in Anhui Province still at a low level of tourism development.

Similarly, the critical values of 0.3, 0.5, and 0.8 were used to classify green development efficiency into four grades, namely, low, medium, high, and higher efficiency (Figure 3b). As shown in Figure 3b, urban green development efficiency in the Yangtze River Delta region was generally not high and tended to decrease. Specifically, the number of cities with high and higher green development efficiency was 10, 9, and 4 in 2000, 2009, and 2018, respectively. The number of cities with low and medium green development efficiency accounted for 75.61%, 78.05%, and 90.24% in 2000, 2009, and 2018, respectively. Overall, the gradual decrease in the number of cities with high and higher green development efficiency implied that the current economic development mode in the Yangtze River Delta region still relied heavily on the consumption of resources and environment, and there was still much room for improving urban green development efficiency. Besides, urban green development efficiency showed a distribution pattern of “high in the south and low in the north”. The cities with high and higher green development efficiency in 2000 were concentrated in southern Zhejiang Province and southwestern Anhui Province and scattered in Shanghai and Huai’an, while the low-value areas were clustered in the northwestern delta and northern Zhejiang Province. In 2009, the high-value areas of green development efficiency were more dispersed, and the low-value areas were still mainly distributed in the northern and central delta. In 2018, cities with high and higher green development efficiency shrank to Huangshan, Shanghai, Hangzhou, and Quzhou, while cities with low green development efficiency tended to be more concentrated in the northern delta.

### 5.2. The Impulse Response Relationship between Urban Tourism Development and Green Development Efficiency

Before testing whether there was a long-run equilibrium and causal relationship between tourism development and green development efficiency, the following tests were required. Firstly, in order to avoid pseudo-regression, the IPS, HT, PP-Fisher, and LLC unit root methods were chosen to test the unit root of urban tourism development and green development efficiency (Table 3). As found in Table 3, urban tourism development and green development efficiency passed the 1% statistical significance level, implying the two systems were time-series smooth. Secondly, Granger causality tests were executed to test the causal relationship between urban tourism development and green development efficiency (Table 4). As shown in Table 4, the hypothesis that urban green development efficiency was not the Granger cause of tourism development could not be rejected at the 10% level of significance. The hypothesis that urban tourism development was not the Granger cause of green development efficiency could be significantly rejected. This indicated that there was a unidirectional Granger causality relationship between urban tourism development and green development efficiency in the Yangtze River Delta region, and tourism development exhibited a positive contribution to the improvement of green development efficiency.

**Figure 4:**
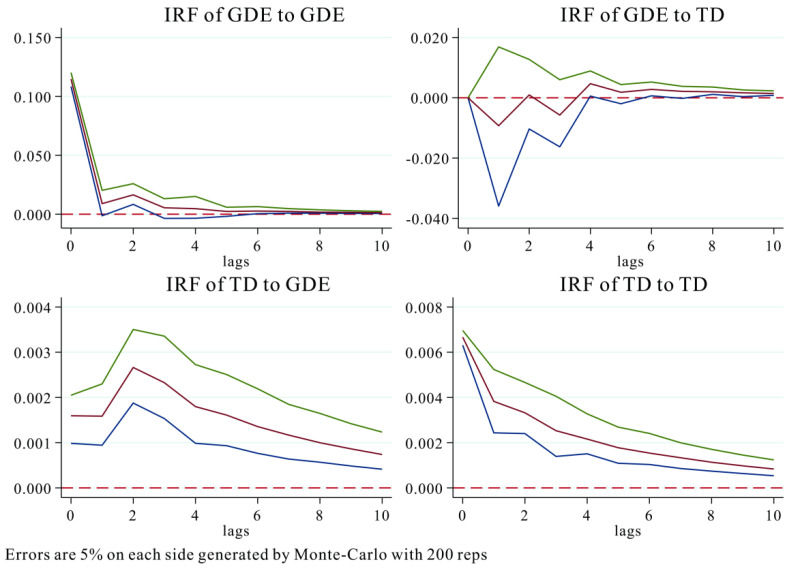
Impulse response relationship between TD and GDE. Note: The red line is the impulse response curve, the green and blue lines represent the 5% and 95% quantile lines.

The impulse response function was further plotted using the PVAR model, and the dynamic shock effect between tourism development and green development efficiency was analyzed after 300 Monte-Carlo simulations (Figure 4). As shown in Figure 4, in terms of the shock effect of GDE on itself and TD, the shock effect of GDE on itself showed some positive responses in periods 1 and 2, then declined rapidly and finally converged to zero from period 3 onwards. For the impulse shocks from TD, GDE showed a negative response with a significant downward peak in period 1, a rapid convergence to 0 in period 2, another significant decline in period 3, and a significant positive response in period 4, which then gradually weakened and converged to 0. This indicated that the impact of GDE on TD exhibited a significant attenuating impact in the short term, while the shock response was not insignificant and converged to zero in the long term. In terms of the shock effect of TD on itself and GDE, the shock effect of TD on GDE was exceptionally significant, and this positive driving effect persisted in the long run. The impact intensity gradually reached the maximum value in period 2, and then the impact effect weakened slowly and approached 0. The response of TD to its own shocks showed a decreasing trend, and the impact intensity was 0.0065 in period 0, and then gradually decreased. This implied that tourism, as a representative of the green industry played an important role in promoting socioeconomic development and energy conservation and emission reduction. With the sustainable development of the tourism economy and the accumulation of wealth, it would have a positive driving effect on green development efficiency.

### 5.3. The Influencing Effects of Tourism Development on Green Development Efficiency

According to existing studies, green development efficiency was also influenced by many other socioeconomic factors, so it was necessary to further identify the impact of TD on GDE while controlling for other relevant variables (Table 5). Thereinto, (1) the level of economic development, as an important component of desirable output, is an important driver of green development and directly affects the efficiency of green development. However, inevitably, the process of economic development also increases the level of resource consumption and environmental pollution [47,48]. Per capita GDP was used to characterize urban economic development level (*ED*). (2) Different industries have significant differences in the scale and efficiency of economic and environmental output, while industrial development has the heaviest impact on resource consumption and environmental pollution and is an important part of the secondary industry [49,50]. The ratio of the industrial-added output value to GDP (%) was used to characterize industrial structure (*IS*). (3) Improvements in advanced production and management technologies can directly contribute to green technological progress and reduce energy consumption and environmental pollution, thus promoting green development efficiency [51]. The energy consumption of 10,000 Yuan GDP was used to represent urban innovation ability (*IA*). (4) There are generally two opposite views about foreign direct investment. On the one hand, economically developed countries transfer highly polluting and energy-intensive industries to underdeveloped regions for production at a lower environmental cost, which exacerbates local resource consumption and environmental pollution and creates a “pollution refuge” effect. On the other hand, foreign direct investment may also bring advanced technology and management concepts, generate technological spillover effects, and stimulate the transformation of local economic development [52,53]. As an important foreign trade agglomeration, the Yangtze River Delta region accounts for one-fourth of the country’s foreign direct investment, thus the ratio of foreign direct investment to regional GDP was used to characterize the level of regional openness to the outside world. (5) Environmental regulation can directly reduce environmental pollution emissions and resource waste through the control and treatment of pollutant sources and production processes, so that economic efficiency can be improved. However, environmental regulation may increase firms’ production costs and induce fluctuations in green development efficiency [54,55]. The ratio of total environmental investment to GDP was selected to measure the intensity of environmental regulation (*ER*). (6) Government policy regulation is an important means to promote energy conservation and emission reduction in the whole society. By formulating corresponding policies of command and control, economic incentives, and public participation, it not only influences the institutional prospect of green technology development, but also effectively promotes enterprises to innovate production technology, reduce pollutant emissions and form a public participatory and conservation-oriented society [56,57]. The proportion of fiscal expenditure to regional GDP was selected to reflect the influence of urban government intervention (*GI*).

The optimal lag period 2 of GDE was determined as the instrumental variable to estimate the SGMM model. To illustrate the validity and robustness of the estimation results, the mixed OLS model and the panel fixed effect model were also estimated by using Stata16.0 software. Moreover, in order to avoid collinearity, the stepwise regression method was used, and relevant variables were successively introduced into 7 groups for regression (Table 6). As shown in Table 6, the coefficient of the lag term of the independent variable was between the coefficient values of the OLS model and the fixed effect model, which indicates that the SGMM model was reasonably set.

The coefficient of tourism development was 0.305 and passed the 1% significance level test, and the coefficient of the quadratic term of tourism development was −0.010 and also passed the 1% significance level test, which indicated that there existed an inverted U-shape relationship between tourism development and green development efficiency. This was due to the fact that the rapid development of the tourism industry played a certain multiplier effect on urban economic growth and job creation, and this positive effect offset the external negative effect, thus significantly promoting green development efficiency. However, with the advent of mass tourism, tourism resources were continuously being developed and the scale of tourism continued to expand, and the negative impact of tourism development on resource consumption, environmental pollution, and local culture gradually appeared. Tourism was no longer a “smokeless” industry, and due to its heavy dependence on oil, coal, and other energy sources, tourism had become a major carbon emitter, which made tourism development produce a negative impact on urban green development efficiency.

The elasticity coefficient of economic development on green development efficiency was 0.055 and passed the 1% significance level test, which indicated that economic development level had a significant positive driving effect on green development efficiency. This was due to the fact that economic development not only promoted the rationalization and advancement of industrial structures and provided a solid material foundation for green growth, but also improved residents’ environmental awareness and demands, thus exerting a positive impact on green development efficiency. However, the economic development mode relying on the input and consumption of natural resources should be avoided, which may increase ecological environment pollution and undesirable outputs and hinder the sustainable and rapid improvement of green growth.

The elasticity coefficient of the proportion of secondary industry was significantly negative, indicating that the industrialization level had a negative driving effect on green development efficiency in the Yangtze River Delta region. This was because the secondary industry had always been a major sector with high resource consumption and high environmental pollution in national economic development. The higher the industrialization rate, the lower the green development efficiency. It could be anticipated that with the development of the social economy, regional cities gradually entering into the post-industrial era, the degree of industrialization would tend to decrease, and industrial production would be cleaner and more efficient, which contributed to improving green development efficiency.

At the 1% significance level, the influence coefficient of innovation capacity on urban green development efficiency was −0.037. The innovation capacity characterized by energy consumption per ten thousand yuan of GDP was an inverse indicator, the higher energy consumption per ten thousand yuan of GDP, the weaker innovation ability, and vice versa, which implied that the stronger the innovation capacity, the higher green development efficiency. This was because technological innovation could effectively promote social development, improve economic production efficiency, optimize industrial structures, transform economic growth mode, and thus improve urban green development efficiency.

The elasticity coefficient of foreign direct investment on urban green development efficiency was −0.02, which passed the 1% level of the statistical significance test. This was because the Yangtze River Delta region was highly open to the outside world and has frequent foreign economic and trade exchanges. Due to the relatively looser environmental access policies, a large amount of global logistics, information flow, and capital flow were concentrated in the Yangtze River Delta region, which accounted for a quarter of China’s foreign investment. A large amount of foreign direct investment not only promoted rapid urban economic development, but also consumed a large amount of natural resources and increased the level of environmental pollution. However, the “pollution halo” effect was offset by the “pollution haven” effect, which would restrict the improvement of green development efficiency.

The influence coefficient of environmental regulation on green development efficiency was 0.003, but not significant. This was due to that environmental regulation could reduce and restrain environmental pollution emissions and resource waste through the long-term investment of capital and technology, and finally improved green development efficiency. However, the enhancement of environmental regulation intensity would lead to the increase in pollution cost of enterprises, inhibit the investment of enterprises in cleaner production technology, and affect the production efficiency and direct economic benefits of enterprises, which was insignificantly conducible to improving green development efficiency in the short term. With the continuous implementation of environmental regulations, the scale and proportion of investment in environmental pollution control would significantly increase, which would lead to the reduction in resource consumption and environmental pollutant emissions and promote the improvement of green development efficiency.

At the 1% level, government intervention had a significant positive impact on green development efficiency. Every 1% increase in the degree of government intervention would improve the green development efficiency of the Yangtze River Delta urban agglomeration by 0.002%. This was because that government regulation was an important driving force to promote social energy conservation and emission reduction. It could effectively promote enterprises to innovate production technology, reduce pollutant emissions, and thus improve green development efficiency by integrating command and control with economic incentive policies.

## 6. Discussion

At present, China’s economy has shifted from the stage of high-speed growth to the stage of high-quality development, the battle against pollution prevention and control has achieved initial results, and the national strategy of “ecological priority and green development” has been steadily implemented and deeply rooted in people’s hearts, and will become the main way and driving force for China’s economy to achieve high-quality development for a long period of time in the future [58]. Under the background of ecological civilization construction and green development, tourism becomes an important carrier to practice the “Two Mountains Theory” and meet people’s ever-growing needs for a better life, and exploring the issue of the green development effect of China’s tourism industry will realize the necessary echo between the development of China’s tourism industry and the national strategy of “ecological priority and green development” [59]. Depending on the long-term sustained growth of China’s macro economy, the promotion of structural reform of the tourism supply side, and the continuous improvement of the tourism consumption environment, the scale of the tourism economy in the Yangtze River Delta region continues to expand and the status of tourism in the national economy continues to strengthen. So, as a “smokeless industry” and a “green industry” from the traditional perspective and cognition, can the development of tourism really effectively promote the green transformation and high-quality development of the economy? This is not only a practical question about the value appeal and realistic mission of tourism development, but also a theoretical question about the demonstration of the green attribute of tourism, which is obviously worthy of necessary attention. Hence, this study introduced tourism economy into the research scope of green development and revealed that urban tourism and green development efficiency tourism development had a one-way interactive relationship, which provides theoretical support and empirical evidence for the causal relationship between tourism economy and green development efficiency and enriches the research scope of green development theory [60]. Moreover, the inverted U-shape impact of the urban tourism economy on green development efficiency in the Yangtze River Delta was found, which broadens the application field of the environmental Kuznets curve theory [61].

The complexity and dynamics of the influence mechanisms of tourism on urban green development efficiency and the heterogeneity of tourism destinations (such as urban spatial heterogeneity, economic heterogeneity, cultural heterogeneity, and endowment heterogeneity, etc.) are the main reasons for the nonlinear effects of tourism’s green development effects [61,62]. On one hand, the development of tourism needs the support of a good ecological environment in tourism destinations, and in the process of tourism development, the environmental protection awareness of the residents in tourism destinations will also be enhanced, and tourism income will also provide financial support for environmental protection [63]. In addition, the tourism industry will promote local economic growth through the effects of the integration of related industries, the replacement of industrial structures, the expansion of opening up to the outside world, and the optimization of the development environment [64]. Hence, tourism, as a pillar industry of regional economic and social development and happiness industry, played an important role in the construction of urban ecological civilization, high-quality development, and promotion of people’s well-being, and contributed to improving urban green development efficiency from the three dimensions of economy, society, and ecology in the short term [65].

However, tourism development after crossing the inflection point will hinder the improvement of urban green development efficiency, which is not conducive to the long-term sustainable development of tourism. This is due to that the development of tourism activities depends on the investment and consumption of capital, human, material, and financial resources in tourism destinations. Tourism development will inevitably result in the depletion of natural capital consumption, and pollutants such as carbon emissions, wastewater, and solid waste generated by tourism activities can also negatively affect the resources and environment of tourism destinations influenced by the “temporal inertia” of ecological environment and the “space path” of industrial interdependence [61]. How to break this situation and sustain the positive driving effect of tourism development needs to be focused on in the future. Firstly, although tourism development can effectively promote urban green development efficiency, it should be fully recognized that tourism is not really a smokeless industry and that the carbon emissions from tourism in existing studies are far more underestimated than the actual carbon emissions [66,67]. Thus, in the context of low-carbon transition, energy conservation and emission reduction and green environmental protection of tourism development should be placed at the forefront of the whole process of ecotourism. Besides, given the linkage drive of the tourism industry and the spatial mobility of tourism activities, it is urgent to construct a research framework on the impact and spatial spillover effects of the tourism economy on the overall green development of cities [68].

## 7. Conclusions

Based on the panel data of 41 prefecture-level cities in the Yangtze River Delta region from 2000–2018, this paper analyzed the spatio-temporal evolutionary and interactive characteristics between tourism development and green development efficiency in Yangtze River Delta cities, and then explored the nonlinear influence effect of tourism development on green development efficiency. The following are the findings of the study.

(1) Tourism development in the Yangtze River Delta cities showed a general W-shaped upward trend from 2000 to 2018. The green development efficiency presented significant phase characteristics with an upward phase from 2000 to 2004, a fluctuating phase from 2004 to 2015, and an inverted “V” shaped saddle phase from 2015 to 2018. In terms of spatial pattern, the tourism economy of most cities in the delta region continued to improve and change from a low level to a high level, and generally showed an overall pattern of “high in the southeast delta and low in the northwest delta”. The overall level of green development efficiency of regional cities was not high, showing a pattern of “high in the south delta and low in the north delta”.

(2) There also was a one-way Granger causality relationship between tourism development and green development efficiency in the Yangtze River Delta region [69,70]. The continuous development of the tourism economy exhibited a positive contribution to the improvement of green development efficiency. Specifically, the impulse response effect of the tourism economy on green development efficiency was exceptionally significant, and this positive driving effect would be sustained and maintained in the long term, while the impact effect of green development efficiency on the tourism economy was only reflected in the weak effect in the short term and not significant in the long term, which further confirmed the positive role of the tourism economy on green development.

(3) There was a significant inverted U-shaped relationship between tourism economy and green development, which was characterized by the nonlinear effect of promoting first and then hindering [61]. The level of economic development and the degree of government intervention had significant positive effects on green development efficiency. Although environmental regulation could promote the efficiency of urban green development, its effect was not significant. In addition, the variables of industrial structure, innovation ability, and foreign direct investment had a significantly negative driving effect on green development efficiency.

## 8. Policy Implications

Based on the empirical results, this study proposes the following policy recommendations:

Firstly, it is necessary to continuously promote the green development of upstream and downstream tourism-related enterprises. Tourism enterprises should actively take the road of digital development, innovate the development mode of ecotourism, explore the value realization mechanism of ecotourism products, and strengthen the guidance and management of ecotourism, so as to make tourism become an important driving force for inclusive growth and environmental sustainability, and comprehensively promote the development of ecotourism.

Secondly, it is necessary to establish the regional tourism innovation chain to form a tourism innovation spillover mechanism, so as to strengthen the radiation driving effect of the tourism economy on green technology progress. With the implementation of the region-wide tourism strategy and the era of cultural and tourism integration, relevant government departments should give full play to the driving, catalytic and integrated role of “tourism +”, cultivate new operational types of tourism, and construct the development pattern of “tourism space all-region, tourism industry all-field, and tourism audience all-people”, so as to enhance the overall innovation capacity of the city, and drive green development through the two wheels of green technology efficiency and green technology progress.

Thirdly, it is necessary to promote regional tourism industry cooperation, build the Yangtze River Delta tourism development community, form a joint prevention and control mechanism for tourism economic externalities and environmental externalities, stimulate the green contribution capacity of the tourism industry, and lead the green integrated development of the Yangtze River Delta. Specifically, local governments should implement regional tourism integration policies, establish inter-regional tourism cooperation mechanisms, optimize the spatial layout of tourism product supply, eliminate barriers to the flow of tourism in the delta region by sharing the source market, sharing human capital, building and sharing supporting infrastructure, co-creating regional tourism routes, etc., give full play to the radiating role of regional tourism growth poles, drive the development of tourism in the surrounding hinterland, and ultimately achieve a regional tourism development pattern of mutual support, healthy competition, and synergistic development

Admittedly, this study could be further advanced from the following aspects. Firstly, although this study conducted a systematic empirical analysis of green development efficiency, the decomposition of pure technical efficiency and scale efficiency terms was not considered. In the future, the network DEA model can be used to divide the green development efficiency into two stages and further investigate the intrinsic influence mechanism of tourism development on green development efficiency. Secondly, this study only explored the direct effect of tourism development on green development efficiency, while ignoring the transmission mechanism of other mediating variables. In the future, the mediating effect model and structural equation model can be used to explore the green development effect of tourism development in depth from the non-linear and multi-scale perspective. Thirdly, the geographically and temporally weighted regression (GTWR) model can be further used to explore the heterogeneous and correlational impact of tourism development on green development efficiency, so as to deepen the scientific understanding and practical guidance on the effect of tourism development on green development efficiency.

## Figures and Tables

**Table 1:** Green development efficiency measurement index system.

Primary Indices	Secondary Indices	Third Indices	Indicators Description
Input indicators	Non-resource inputs	Capital input	Total investment in fixed assets (billion yuan)
		Labor input	Urban employees (10,000 people)
		Technical input	The number of patent licenses applied for in various places (pieces)
	Resource inputs	Energy and land resource consumption	Construction land area (km^2^), total energy consumption (million tons of standard coal)
Output indicators	Desirable outputs	Economic benefit	GDP (billion yuan)
		Social benefits	Total retail sales of consumer goods (billion yuan), number of doctors per 10,000 people
		Ecological benefit	Green space area (km^2^), air quality rate (%)
	Undesirable outputs	Environmental pollution	Industrial wastewater emissions (million tons), industrial waste gas emissions (million m^3^), industrial solid waste emissions (million tons)

**Table 2:** Tourism development evaluation index system.

Target Layer	Guideline Layer	Indicator Layer	Unit
Tourism development level	Tourism scale	Total tourism revenue	Million yuan
		Number of visitors	Million people
		Number of travel agencies	Number
	Tourism quality	Per capita domestic tourism consumption expenditure	Yuan
		Per capita inbound tourism consumption expenditure	Dollar
		Number of scenic spots above 3A level	Number
	Tourism structure	Number of star-rated hotels	Number
		The proportion of tourism in the tertiary sector	%
		The share of tourism to GDP	%

**Table 3:** Results of unit root test for TD and GDE.

Test Method	IPS Test		HT Test		PP-Fisher Test		LLC Test		Judgment Result
	Z-Value	*p*-Value	Z-Value	*p*-Value	Z-Value	*p*-Value	Z-Value	*p*-Value	
*lnGDE*	−8.716	0.000	−21.679	0.000	−13.252	0.000	−10.579	0.000	stable
*lnTD*	−3.909	0.000	−11.645	0.000	−4.056	0.000	−3.980	0.000	stable

**Table 4:** Results of Granger causality test for TD and GDE.

Equation	chi2	Prob > chi2	Conclusion
*lnGDE* is not the Granger reason of *lnTD*	0.623	0.430	Accept the original hypothesis
*LnTD* is not the Granger reason of *lnGDE*	3.323	0.068	Reject original hypothesis *

Note: * in the table indicate rejection of the original hypothesis at 10% significance level.

**Table 5:** Selection of indicators for green development efficiency impact factors.

Control Variable	Symbol	Unit	Calculation Method
Economic development	*ED*	yuan	Per capita GDP
Industrial structure	*IS*	%	Industrial added value/GDP
Innovation ability	*IA*	Ton standard coal/ten thousand yuan	Energy consumption per 10,000 yuan GDP
Foreign direct investment	*FDI*	%	Foreign direct investment/GDP
Environmental regulation	*ER*	%	Total environmental investment /GDP
Government intervention	*GI*	%	Financial expenditure /GDP

**Table 6:** Systematic dynamic panel regression results of the factors influencing green development efficiency.

Variables	OLS Model	Fixed Effect Model	SGMM Model						
			Model 1	Model 2	Model 3	Model 4	Model5	Model 6	Model 7
*GTFP*_1	0.042	0.031	0.088 ***	0.072 ***	0.073 ***	0.067 **	0.061 ***	0.035	0.029
	(1.13)	(0.98)	(3.98)	(3.48)	(2.95)	(2.53)	(2.88)	(1.02)	(0.95)
*lnTE*	0.302 ***	0.334 ***	0.424 ***	0.424 ***	0.417 ***	0.346 ***	0.312 ***	0.317 ***	0.305 ***
	(4.93)	(3.74)	(28.77)	(24.00)	(17.08)	(12.03)	(8.16)	(9.16)	(9.72)
*lnTE*_2	−0.020 ***	−0.011 ***	−0.013 ***	−0.013 ***	−0.013 ***	−0.011 ***	−0.010 ***	−0.010 ***	−0.010 ***
	(−4.46)	(−3.50)	(−25.87)	(−23.63)	(−15.51)	(−11.62)	(−7.49)	(−8.28)	(−8.70)
*lnED*	0.056 ***	0.058 ***		0.029 ***	0.037 ***	0.035 ***	0.032 ***	0.027 **	0.055 ***
	(3.95)	(8.95)		(3.17)	(4.15)	(3.51)	(3.03)	(2.17)	(10.51)
*lnIS*	−0.054	−0.079 **			−0.087 **	−0.041	0.014	0.010	−0.072 **
	(−0.74)	(−2.17)			(−2.39)	(−1.09)	(0.32)	(0.21)	(−2.41)
*lnIA*	−0.035 **	−0.036 ***				−0.045 ***	−0.042 ***	−0.045 ***	−0.037 ***
	(−2.51)	(−2.99)				(−6.88)	(−4.57)	(−4.97)	(−4.01)
*LnFDI*	−0.002 **	−0.002 ***					−0.001 ***	−0.002 ***	−0.002 ***
	(−2.32)	(−6.06)					(−8.17)	(−6.10)	(−4.64)
*lnER*	0.008	0.007						0.007	0.003
	(1.27)	(1.17)						(1.16)	(0.52)
*lnGI*	0.002 ***	0.001 ***							0.002 ***
	(3.23)	(3.70)							(3.96)
*_cons*	−1.952 ***	−2.189 ***	−2.533 ***	−2.734 ***	−2.703 ***	−2.093 ***	−1.892 ***	−1.869 ***	−1.957 ***
	(−4.40)	(−3.33)	(−24.11)	(−21.01)	(−16.06)	(−10.82)	(−6.66)	(−7.10)	(−8.34)

Note: ** and *** indicate significant at 0.05 and 0.01 levels, respectively, with t-statistics in parentheses.

## Data Availability

Data available from the authors upon request.
